# Esophageal motility, vagal function and gastroesophageal reflux in a cohort of adult asthmatics

**DOI:** 10.1186/1471-230X-12-140

**Published:** 2012-10-12

**Authors:** D Lakmali Amarasiri, Arunasalam Pathmeswaran, Anuradha S Dassanayake, Arjuna P de Silva, Channa D Ranasinha, H Janaka de Silva

**Affiliations:** 1 Department of Physiology, Faculty of Medicine, University of Kelaniya, Ragama, Sri Lanka; 2 Department of Public Health, Faculty of Medicine, University of Kelaniya, Ragama, Sri Lanka; 3 Department of Pharmacology, Faculty of Medicine, University of Kelaniya, Ragama, Sri Lanka; 4 Department of Medicine, Faculty of Medicine, University of Kelaniya, Ragama, Sri Lanka

## Abstract

**Background:**

Asthmatics are known to have esophageal hypomotility. Vagal hypofunction and prolonged intra-esophageal acidification cause esophageal hypomotility. The contribution of gastroesophageal reflux (GER) and vagal function to esophageal motility in asthmatics is unclear. We studied the relationship between esophageal motility, GER and vagal function in a cohort of adult asthmatics.

**Methods:**

Thirty mild, stable asthmatics (ATS criteria) and 30 healthy volunteers underwent 24-hour ambulatory esophageal monitoring, manometry, autonomic function testing and GER symptom assessment. 27 asthmatics underwent gastroscopy. A vagal function score calculated from 3 tests (valsalva maneuver, heart rate response to deep breathing and to standing from supine position) was correlated with esophageal function parameters.

**Results:**

Asthmatics (mean age 34.8 (SD 8.4), 60% female) had more frequent GERD symptoms than controls (mean age 30.9 (SD 7.7), 50% female). 10/27 asthmatics had esophageal mucosal damage, 22 showed hypervagal response, none had a hyperadrenergic response. 14 asthmatics had ineffective esophageal motility. Higher GERD-score asthmatics had significantly fewer peristaltic and more simultaneous contractions than controls, and higher esophageal acid contact times than those with lower scores. All reflux parameters were significantly higher and acid clearance time prolonged in asthmatics than controls (p < 0.001, Mann–Whitney U test). There was no correlation between vagal function score and esophageal function parameters.

**Conclusions:**

A cohort of adult asthmatics was found to have peristaltic dysfunction and pathological GER, but otherwise normal esophageal motility. The peristaltic dysfunction seems to be associated with vagal hyperreactivity rather than vagal hypofunction.

## Background

Asthmatics are known to have an increased prevalence of gastroesophageal reflux disease (GERD) symptoms, esophageal hypomotility and abnormal acid exposure [1].

A study revealed that asthmatics had significantly lower LES pressures [1]. In 34 non-allergic asthmatic people who underwent esophageal manometry because of gastrointestinal symptoms, 23 (68%) had esophageal dysmotility with low amplitude esophageal contractions, hypotensive lower esophageal sphincter (LES) and diffuse esophageal spasm [2]. Ineffective esophageal motility (IEM) was found to be the most common motility abnormality in patients with GERD-associated respiratory symptoms [3]. Studies on asthmatics revealed increased frequency of transient lower esophageal sphincter relaxations (TLESRs) and increased acid contact times [4].

Reduced motility of the esophagus may be due to vagal hypofunction [5]. However on the contrary asthmatics have evidence of a hypervagal response [6]. A study on 15 asthmatics revealed that 8 had a hypervagal response and of the 7 who had a mixed response there was a predominance of hypervagal tone [7].

Esophageal hypomotility may also be secondary to damage from prolonged intra-esophageal acidification. Abnormal reflux is prominent in asthmatics. An endoscopic study of 156 adult asthmatics showed that 39% had evidence of esophagitis or Barrett esophagus [8]. A retrospective analysis of 24 hour pH monitoring done in 199 asthmatics showed that 82% had symptomatic GER, of which 72% were reflux positive [9]. Sontag *et al.* reported that 82% of 104 adult asthmatics had abnormal amounts of acid reflux, more frequent reflux episodes, higher esophageal contact times and longer esophageal acid clearance times (p < 0.0001 for all parameters tested) irrespective of presence of symptoms [1]. In 105 consecutive asthmatics, the prevalence of GER was 32% on prolonged ambulatory pH monitoring [10].

Hence, whether esophageal hypomotility in asthmatics is due to vagal hypofunction or secondary to damage from gastro-esophageal reflux (GER) is unclear. The aim of this study was to study the relationship between esophageal motility, GER and vagal function in adult asthmatics.

## Methods

Thirty consecutive asthmatics who met American Thoracic Society (ATS) guidelines for the definition of asthma (12% improvement and 200 mL increase in FEV_1_ following bronchodilator administration) [11] were recruited from medical clinics, irrespective of their GERD symptom status, asthma severity or asthma medication use. Controls were either clinic attendees, those accompanying them or hospital staff who denied having respiratory symptoms, asthma or other respiratory illness. Being a smoker, alcoholic, diabetic, presence of known esophageal disease or a history of previous upper gastro-intestinal surgery served as additional exclusion criteria for both asthmatics and controls.

This study was approved by the Ethics and Scientific Review Committee of the Faculty of Medicine of the University of Kelaniya, Ragama, Sri Lanka. All procedures were conducted following written informed consent and conform to the Declaration of Helsinki.

All subjects underwent autonomic and esophageal function tests, a GERD symptom assessment and the asthmatics underwent upper gastro-intestinal endoscopy.

### Vagal autonomic function was assessed by

a) valsalva maneuver produced by sustaining a forced expiration through a mouthpiece connected to a manometer (40 mmHg) lasting 15 s, following a deep inspiration

b) heart rate variation with quiet and deep breathing (six breaths per minute)

c) heart rate response to standing from supine position.

Sympathetic function was assessed by:

d) blood pressure response to standing from supine position.

For correlation with gastro-intestinal function parameters, a vagal function score was calculated from the results of the three tests. For each test, the result of each individual was ranked on a scale from 1 to 100. The three values were added and averaged for an individual.

### GERD symptom assessment

Both asthmatics and controls were screened by a previously validated GERD screening score assessing frequency and severity of seven common upper gastrointestinal symptoms on a 5-point Likert scale. Symptoms included were 1) heartburn 2) regurgitation 3) upper abdominal or chest pain 4) abdominal distension 5) dysphagia 6) cough and 7) belching. A GERD score was calculated as the sum of the products of frequency and severity of each symptom. A cut of score of ≥ 12.5 was considered as a positive GERD score [12].

### Upper gastrointestinal (UGI) endoscopy

UGI endoscopy was performed in 27 asthmatics that consented. The procedure was performed by 2 trained physicians using a fibreoptic endoscope (Olympus, CLV-U20) and findings graded according to modified Savary-Miller criteria [13]. Endoscopy was not performed in controls.

### Tests of esophageal function

Esophageal motility was assessed by the stationary pull-through method, using a water perfused system (Synetics PC Polygraf system, Stolkholm, Sweden) according to standard methodology. We had no provision to measure LES pressure using a Sleeve mechanism.

Antacids were discontinued 24 hours before, drugs that could affect gastrointestinal motility at least 48 hours before and any anti-secretory drugs one week prior to the study. All asthma medication was withdrawn for 48 hours, allowing inhaled beta-2 agonists on an as needed basis. No food or drink was allowed for at least 6 hours prior to intubation to reduce risk of aspiration during intubation [14].

Nasal intubation was done with application of local anesthetic gel (lignocaine) and a lubricant gel. LES, esophageal body and UES pressures were studied according to standard pull-through method in response to 5 mL water boluses. Overall parameters were summated for each subject to classify them manometrically [15].

24-hour ambulatory esophageal pH monitoring was performed on all subjects using a dual sensor monocrystalline antimony catheter (Synetics Medical AB, Stolkholm, Sweden), according to standard methodology [16]. The distal sensor of the pH catheter was positioned 5 cm above the superior border of the manometrically determined LES and the proximal sensor was 15 cm above the distal sensor. Reflux was assessed based on the score derived from DeMeester and Johnson, namely: total number of reflux episodes, duration of the longest reflux episode, number of reflux episodes lasting longer than 5 minutes, total percentage of time pH < 4, time pH < 4 during upright exposure, time pH < 4 during supine exposure and the DeMeester score. The acid contact time was defined as the percentage of the total 24 hour period with pH < 4. Meal times, supine periods, and GERD symptoms during the recording period were noted for each subject [17].

### Statistical analysis of results

Non parametric tests (Wilcoxon Rank Sum test for paired data and Mann Whitney U test for unpaired data) were used to compare the data between the asthmatics and controls. Data was expressed as median (range) using 5^th^ and 95^th^ centile values unless specified otherwise. The upper 95^th^ centile of normal values was used to classify abnormal pH exposure. Categorical variables were compared using the χ^2^ and Fisher Exact tests. Spearman rank correlation was used to study the association between vagal function and esophageal motility and 24 hour ambulatory pH monitoring variables. A *P* value of ≤ 0.05 was considered significant. All statistical analysis was performed using SPSS version 10.0 for Windows software (SPSS Inc., Chicago, IL, USA).

## Results

### Demographics

The 30 asthmatics and 30 controls that completed the study were found to be comparable in terms of age, gender and body mass index. The asthmatics had a higher frequency and severity of GERD symptoms (Table 1).

**Table 1:** Baseline characteristics of asthmatics and controls

	***Controls***	***Asthmatics***	***P value***
Age, yrs	30.9 ± 7.7	34.8 ± 8.4	0.075
Gender, (M:F)	15:15	12:18	0.604
BMI, (kg/m^2^)	21.8 ± 3.6	21.3 ± 4.4	0.647
GERD symptom score (frequency x severity)	7.6 ± 0.8	32.0 ± 21.1	0.000^*^

^*^ Student t-test BMI = Body Mass Index GERD = gastro-esophageal reflux disease.

All values mean ± SD unless specified otherwise.

All asthmatics had only occasional asthma symptoms over the preceding two weeks before the study. They continued their current medication (Table 2). On GERD symptom assessment, all controls had negative GERD symptom scores. Of the asthmatics, 20 had positive GERD symptom scores and 10 had negative GERD symptom scores. The demographic details of the asthmatic population are given in Table 2. The two groups differed significantly only in the mean GERD symptoms scores and presence of esophagitis.

**Table 2:** Demographic details of asthmatics

	***Asthmatics with negative GERD symptom scores (n = 10)***	***Asthmatics with positive GERD symptom scores (n = 20)***	***All asthmatics (n = 30)***
Age, yrs mean ± SD	35.4 ± 9.6	34.5 ± 8.0	34.8 ± 8.4
Gender, (M:F)	2:8	9:11	12:18
BMI, (kg/m^2^), mean ± SD	20.3 ± 2.4	21.9 ± 5.1	21.3 ± 4.4
GERD symptom score, mean ± SD*	10.0 ± 1.6	43.1 ± 17.0	32.0 ± 21.1
Upper GI endoscopy status, no of subjects ^**^			
Normal	5	12	17
Esophagitis, grade 1	2	7	9
Esophagitis, grade 2	-	-	-
Esophagitis, grade 3	-	1	1
Severity of asthma, no of subjects (%)			
Mild intermittent	10 (100)	16 (80)	26 (87)
Mild persistent	-	4 (20)	4 (13)
On oral drugs, no of subjects (%)			
Oral salbutamol	6 (60)	11 (55)	17 (57)
Inhaled salbutamol	4 (40)	5 (25)	9 (30)
Oral theophylline	5 (50)	6 (30)	11 (37)
Oral steroids	4 (40)	7 (35)	11 (37)
Inhaled steroids	4 (40)	10 (50)	14 (47)
Spirometry results, mean ± SD			
FVC	3.2 ± 0.4	3.2 ± 0.6	3.2 ± 0.5
FEV1	2.7 ± 0.5	2.7± 0.4	2.7 ± 0.4
FEV_1_/FVC %	85.2 ± 9.5	82.9 ± 11.0	83.7 ± 10.4

* p < 0.001; Student t test ** p < 0.05; Fisher exact test.

All other parameters did not significantly differ between the two groups.

Twenty seven asthmatics consented to UGI endoscopy. Of 20 asthmatics with positive GERD symptom scores, 12 had no evidence of mucosal damage, 7 had grade I esophagitis and one subject had grade III esophagitis. Of 7 asthmatics with negative GERD symptom scores, 5 had no evidence of mucosal damage and 2 subjects had grade I esophagitis.

The test values of 29 of the controls were within the normal ranges defined for tests of cardiovascular autonomic function [18]. One demonstrated a hypervagal response. Of the asthmatics twenty two (69%) demonstrated a hypervagal response. None showed a hyperadrenergic response.

### Stationary esophageal manometry study

During the stationary esophageal manometry study, the results of 1 asthmatic and 10 control subjects was excluded from analysis, as equipment failure prevented the manometry study from being performed in these subjects. In those subjects, the position of the lower esophageal sphincter was located by the pH step-up method.

According to the current classification of esophageal motility patterns [15], 16 controls had evidence of normal esophageal motility while 4 showed ineffective esophageal motility. Of the asthmatics, 10 had a normal motility pattern, 2 had evidence of a hypercontracting esophagus and 17 of a hypocontracting esophagus (of which 14 showed IEM, 2 showed a hypotensive LES and 1 had an incompletely relaxing LES).

There was no difference of LES mean end-expiratory values and LES relaxation between asthmatics and controls. There was no difference in the LES length or in UES parameters between the two groups. There was no significant difference in the mean peristaltic amplitude and duration for each group at each position (5 cm, 10 cm and 15 cm above the LES) in the esophagus and the mean peristaltic wave velocity between the two groups (Table 3). When the median percentage of each type of contraction (peristaltic, simultaneous and low amplitude) was compared between the two groups, percentage of peristaltic contractions was significantly lower in the 29 asthmatics compared to the 16 controls who had normal esophageal manometry (80% (10-100%) versus (100% (90–100); *P* = 0.044, Mann Whitney U test). When the data of the 4 controls with IEM was considered, the difference remained, though not significant. Asthmatics with positive GERD symptom scores had a significantly lower percentage of peristaltic contractions and a significantly higher percentage of simultaneous contractions than the controls (Table 3). There was no significant difference in any parameter between controls and asthmatics with negative GERD symptom scores (*P* > 0.05 for all parameters; Mann Whitney U Test).

**Table 3:** Stationary esophageal manometry results of the esophageal body in asthmatics and controls

	***Mean***	***Amplitude***			Waves (%)*			**Velocity of a contraction (cm/ sec)**	**Duration of a contraction (sec)**
	**Total**	**Proximal (5 cm)**	**Mid (10 cm)**	**Distal (15 cm)**	**Peristaltic**	**Simultaneous**	**Low amplitude**		
Controls (n = 16)	74.6 ± 6.3	74.6 ± 7.6	74.9 ± 8.8	74.2 ± 8.9	100 (90–100)	0	0 (0–10)	3.7 ± 0.6	2.5 ± 0.2
Asthmatics with-GERD symptom scores (n = 9)	68.9 ± 7.9	62.7 ± 9.0	63.3 ± 7.0	80.7 ± 3.9	100 (30–100)	0	0 (0–70)	1.8 ± 0.3	6.6 ± 3.9
Asthmatics with + GERD symptom scores (n = 20)	74.6 ± 7.0	57.3 ± 5.6	75.5 ± 8.0	91.2 ± 11.6	75 (10–100)	0 (0–49)	0 (0–70)	4.0 ± 0.8	3.3 ± 0.3
All asthmatics (n = 29)	72.8 ± 5.3	59.6 ± 4.0	71.7 ± 6.0	87.9 ± 9.0	80 (15–100)^†^	0 (0–55)	0 (0–80)	3.3 ± 0.6	4.3 ± 1.2

GERD = gastro-esophageal reflux disease; Values are mean ± SE unless otherwise specified; * Median (range as 5^th^ and 95^th^ centiles);

^†^ p < 0.05, Mann Whitney U test.

### 24 hour ambulatory pH monitoring

All reflux parameters were found to be significantly higher in asthmatics compared to controls (Table 4). Asthmatics with positive GERD symptom scores showed higher total and upright esophageal acid contact times in the proximal esophagus compared to those with negative scores. Abnormal proximal acid reflux was documented in 20 of 30 asthmatics (66.7%) and abnormal distal reflux in 22 of 30 asthmatics (73.3%).

**Table 4:** Acid exposure values among asthmatics and controls

	***Controls***	***Asthmatics with (−) GERD symptom score***	***Asthmatics with (+) GERD symptom score***	All asthmatics*
**Proximal sensor**	**(n = 16)**	**(n = 10)**	**(n = 20)**	**(n = 30)**
Total time pH < 4 (%)	0.07 (0–0.4)	2.0 (0.2-10.6)	2.3 (0.03-50.9)	2.1 (0.04-29.7)
Upright time pH < 4 (%)	0.02 (0–0.4)	1.3 (0–24.0)	3.3 (0–66.3)	1.3 (0–44.0)
Supine time pH < 4 (%)	0.12 (0–0.5)	3.2 (0–17.8)	0.2 (0–34.6)	0.4 (0–25.9)
Total no. of reflux episodes	6.0 (0–25.0)	52.0 (3.0-193.0)	55.5 (0.2-233.0)	52 (1.6-233.0)
No. of episodes ≥ 5 min	0 (0–1.0)	2 (0–128.3)	3.5 (0–122.5)	3 (0–128.3)
Longest episode (min)	0.9 (0–7.8)	10.0 (1.0-155.4)	37.8 (0.06-205.7)	25.8 (0.07-189.4)
DeMeester score	0.95 (0.2-4.8)	23.4 (4.4-64.0)	22.3 (0.2-152.7)	23.4 (0.4-106.0)
**Distal sensor**	**(n = 26)**			
Total time pH < 4 (%)	0.4 (0.08-1.5)	6.3 (1.0-14.7)	7.9 (0.3-35.7)	7.4 (0.5-19.8)
Upright time pH < 4 (%)	0.2 (0–3.0)	6.5 (0–28.8)	4.0 (0–53.9)	4.9 (0.08-30.2)
Supine time pH < 4 (%)	0.4 (0.07-2.8)	4.6 (0.4-13.4)	2.7 (0.01-25.5)	3.0 (0.4-15.6)
Total no. of reflux episodes	18.0 (2.7-94.2)	33.5 (9.0-162.0)	65.5 (10.0-180.0)	43.5 (10.3-162)
No. of episodes ≥ 5 min	0 (0–2)	2.0 (1.0-9.0)	6.5 (0–15.7)	3.5 (1–9.4)
Longest episode (min)	1.9 (0.5-20.6)	12.8 (7.8-104.5)	27.5 (0.5-126.0)	20.1 (1.4-104.5)
DeMeester score	3.35 (0.7-11.5)	17.9 (5.4-55.6)	33.7 (2.2-131.0)	23.1 (5.4-61.2)

Values are median (range as 5^th^ and 95^th^ centiles).

Asthmatics had significantly prolonged proximal and distal acid clearance time (total time pH < 4 divided by the no of reflux episodes) compared to controls (p < 0.001, Mann–Whitney U test). The median acid clearance time values in asthmatics with positive GERD symptom scores was higher than those who had negative GERD symptom scores, but the difference was not significant. The GERD score showed significant negative correlation with LES length and significant positive correlation with the percentage time pH < 4 in the upright position in both proximal and distal positions and the duration of the longest reflux at the proximal sensor. The score also showed an expected negative correlation with amplitude of contractions and UES pressure and expected positive correlations with all the other proximal and distal reflux parameters, though not significant.

## Discussion

Asthmatics are known to have a high prevalence of GER and GER is known to trigger asthma [19]. Initial episodes of reflux may induce acute esophageal injury resulting in lowered LES pressure, delayed acid clearing and more reflux [20]. As reflux continues, aspiration may follow causing the first asthmatic episode. Sensitization of the pulmonary tree may cause the airways to become reactive to other stimuli resulting in bronchospasm through a vagal mechanism [1].

Factors that may promote GER in asthma include, physical characteristics of the individuals (age, gender and body mass index), disruption of LES mechanism (related to asthma severity and presence of hiatal hernia), reduced LES pressure and increased esophageal acid contact times (effect of asthma medication) [21] and autonomic dysregulation [7]. Increasing age [22], the male sex [23] and increased body mass index [24] predispose towards increased GER. The population of asthmatics and controls in the present study was comparable in these characteristics; hence any confounding influence of these factors would have been minimal.

In this study, the asthmatics demonstrated a high use of oral medication (Table 1). Regression analysis revealed that the use of asthma medication (overall and oral versus inhaled) did not seem to influence the difference in oesophageal function parameters between asthmatics and controls.

The asthmatics were recruited from medical clinics. In their history on recruitment it was clarified that these were patients who had been having asthma for a considerable period of time and even childhood asthma. They did not have long term symptoms of gastro-oesophageal reflux disease. The classification regarding presence or absence of GERD symptoms was based on a questionnaire with a recall period of 4 weeks. However, there is no possibility of excluding ‘silent gastro-oesphageal reflux’. So there is a possibility that some may have had GERD as their primary pathology.

The present study revealed that 10 of 27 asthmatics (37%) had evidence of esophagitis. The average prevalence of erosive esophagitis in asthmatics reported in a recent systematic review was also 37% [19]. Most studies report that the severity of esophagitis has a positive correlation with the degree of asthma severity [8,25,26]. The fact that our population of asthmatics had less severe asthma may have contributed to the low prevalence of esophagitis in them. We did not perform UGI endoscopy on the controls. We considered it unethical to do so, when they scored negative for GERD on a previously validated GERD screening questionnaire [12] which was shown to have good correlation with reflux status as determined by 24 hour pH monitoring.

Twenty two (73.3%) asthmatics had abnormally high acid exposure of the distal esophagus and 20 (66.7%) of the proximal esophagus. Individual pH monitoring parameters were significantly higher (both proximal and distal) in asthmatics compared to controls. The acid exposure values were higher in asthmatics that scored positive for GERD symptoms compared to those with lower values. Other studies in similar settings to ours (cross-sectional surveys in secondary care units sampling consecutive asthmatics) have reported high acid exposure values ranging from 14.8-81.8%. A systematic review of these studies revealed a pooled sample-size weighted average prevalence of 50.9% [19]. Of the studies describing dual-sensor pH monitoring, abnormally high proximal acid exposure was reported as 46% in 56 asthmatics [27] and abnormally high distal acid exposure was reported in 78% of 54 asthmatics [28]. Gustafsson *et al.* reported pathological reflux in 50% of asthmatic children in the proximal esophagus and in 16% in the proximal esophagus [29]. DeMeester et al. reported that 50% of patients with chronic respiratory symptoms with proximal reflux had esophageal dysmotility [30].

The anti-reflux barriers that protect against reflux are the gastresophageal junction (LES function), esophageal body motor function and acid clearance and the UES [31].

Asthmatics have been shown to have lower LES pressures [1,32]. The present study showed that only 2 asthmatics had LES hypotension and that the LES pressure value did not differ between asthmatics (with or without reflux) and controls significantly. A previous study reported similar findings [33]. Two studies involving patients with suspected laryngeal reflux have also revealed the same results [34,35]. Stationary pull-through method was employed according to the available facilities in our unit. The gold standard for measurement of LES pressure is use of a Dent Sleeve [36,37]. However this technique was not available to us at the time of the study. Hence interpretation of data regarding LES pressure is done with caution. Failure to demonstrate a relationship between LES pressure and LES events with the severity of GERD could be attributed to the inferior technique employed. This is a severe limitation of the study. With proper and better techniques, a repeated study would enable a more thorough and accurate assessment of actual LES events during reflux episodes in asthmatics.

Another disadvantage in our methodology and hence a major limitation in our study is that stationary pull-through manometry does not record LES pressure continuously, hence fails to capture sphincter relaxations and intermittent reflux episodes.

The UES parameters in our study were similar in asthmatics and controls. Ours was not an ideal setup to measure UES pressure. This is a major limitation in our study, hence data on UES measurement is not discussed here.

Esophageal peristalsis [38] and amplitude of peristaltic contractions [39] play a major role in esophageal acid clearance following GER episodes. Esophageal motility disturbances could contribute to or result from pathological GER. We compared esophageal motility parameters between asthmatics and controls, between asthmatics who had positive GERD symptom scores with those with negative scores and each of these groups separately with controls. When individual esophageal motility parameters were compared [40], asthmatics differed from controls only by a significantly lower proportion of peristaltic waves. Asthmatics with higher GERD symptom scores had lower peristaltic activity.

In the present study the asthmatics demonstrated low peristaltic activity but otherwise normal esophageal manometry. Hence, it is likely that these asthmatics do not have an intrinsic esophageal motility abnormality leading to GER. Therefore, rather than vagal hypofunction contributing to the esophageal dysmotility, as is currently accepted, it may be vagal-hyperreactivity that leads to esophageal dysmotility. Our results lead us to speculate whether this is due to vagal hyperreactivity-induced increased secretion. Whether the reduced esophageal motility is secondary to increased GER due to vagal hyperreactivity and resulting reduced acid clearance warrants further study.

When esophageal parameters were grouped according to motility patterns, asthmatics were found to have a higher frequency of abnormal motility patterns. The most common motility pattern among asthmatics was ineffective esophageal motility (IEM) 44.4% of 29 asthmatics). IEM has previously been described as the commonest motility pattern in subjects with reflux-associated respiratory symptoms [3]. Campo et al. described esophageal dysmotility in 68% of asthmatics with reflux symptoms [2]. Abnormal esophageal motility has been described in patients with chronic cough [41]. Patti et al. found non-specific esophageal motility abnormalities in patients with pulmonary aspiration and GERD [42]. A study from Taiwan reported that out of 56 clinically stable asthmatics, 23 had IEM and 12 had non-specific motility disorder [43]. There are however some studies contradicting this relationship, and these have reported that IEM has no association with GERD or extra-esophageal GERD [44,45].

IEM is associated with increased acid clearance times in the distal esophagus [46]. Esophageal acid clearance time is the amount of time necessary to return the esophagus to a neutral pH following an acidic reflux event [34]. It is one of the important mechanisms that prevent GERD [29]. Initially there is rapid clearance of the refluxate by gravity and primary or secondary peristalsis, followed by slow neutralization of the acid by the swallowed saliva. It is generally assumed that the latter is constant among subjects [34]; hence acid clearance is considered a good measure of esophageal function. Peristaltic dysfunction could delay esophageal acid clearance. This would keep acid in the esophagus for a longer duration and promote acid reaching a higher level [45,47].

Vagal hypofunction is known to contribute to esophageal hypomotility [5]. Studies have reported that in GERD patients, there is no correlation between autonomic function state and esophageal motility or esophageal acid exposure [5]. In the present study, asthmatics did not have evidence of vagal hypofunction, but rather showed a vagal hyper-reactivity. We could not demonstrate correlation between a derived vagal function score and esophageal motility, increased acid exposure or the presence of esophagitis.

The current Montreal consensus on GERD states that though frequency and severity of symptoms have been shown to have a moderate correlation with severity of endoscopic findings in several studies, symptoms cannot accurately predict endoscopic findings of an individual patient [48]. In this context there is difficulty in diagnosing pathological GER by either symptoms or investigation alone. The present study too failed to demonstrate an association of reflux status with upper GI endoscopy findings, symptom severity or esophageal motility pattern among asthmatics.

From the present study findings it seems that hyper-vagal response and prolonged esophageal acid exposure are the more likely reasons for the esophageal peristaltic dysfunction. Increased vagal stimulation increases rate and amount of acid secretion and could therefore augment the damaging effects of GER in these subjects [49]. Acid also inhibits the vagal low threshold mechano-sensors in the esophagus that are responsible for the reflex regulation of esophageal motor functions. This inhibition could result in reduced esophageal clearance or reduced LOS function, thereby favouring further GER [50].

## Conclusions

In conclusion, the present study showed that asthmatics with mild, clinically stable asthma have peristaltic dysfunction and increased GER, but otherwise normal esophageal motility parameters compared to healthy controls. Those with more severe GERD symptoms had more peristaltic dysfunction. The asthmatics also showed a vagal hyper-reactivity rather than a vagal hypofunction. Whether the peristaltic dysfunction is secondary to increased GER resulting from the vagal-hyperreactivity warrants further study.

## Abbreviations

ATS: American Thoracic Society; FEV_1_: Forced expiratory volume in first second; GER: Gastro-esophageal reflux; GERD: Gastro-esophageal reflux disease; IEM: Ineffective esophageal motility; LES: Lower esophageal sphincter; TLESR: Transient lower esophageal sphincter relaxation; UGI: Upper gastro-intestinal.

## Competing interests

The authors have no competing interests.

## Authors’ contributions

All authors were involved in study conception, design, manuscript drafting and revision. AP DS and ASD conducted upper gastro-intestinal endoscopies in asthmatics. DLA and AP were involved in statistical analysis. DLA carried out data acquisition. All authors read and approved the final manuscript.

## Pre-publication history

The pre-publication history for this paper can be accessed here:

http://www.biomedcentral.com/1471-230X/12/140/prepub
